# Preliminary Study of UAS Equipped with Thermal Camera for Volcanic Geothermal Monitoring in Taiwan

**DOI:** 10.3390/s17071649

**Published:** 2017-07-18

**Authors:** Shih-Hong Chio, Cheng-Horng Lin

**Affiliations:** 1 Department of Land Economics, National Chengchi University, No. 64, Sec. 2, ZhiNan Rd., Wenshan District, Taipei 11605, Taiwan; 2 Institute of Earth Sciences, Academia Sinica, No. 128, Sec. 2, Academia Road, Nangang, Taipei 11529, Taiwan; lin@earth.sinica.edu.tw

**Keywords:** unmanned aircraft system (UAS), thermal camera, post-processed kinematic (PPK), volcanic geothermal monitoring

## Abstract

Thermal infrared cameras sense the temperature information of sensed scenes. With the development of UASs (Unmanned Aircraft Systems), thermal infrared cameras can now be carried on a quadcopter UAV (Unmanned Aircraft Vehicle) to appropriately collect high-resolution thermal images for volcanic geothermal monitoring in a local area. Therefore, the quadcopter UAS used to acquire thermal images for volcanic geothermal monitoring has been developed in Taiwan as part of this study to overcome the difficult terrain with highly variable topography and extreme environmental conditions. An XM6 thermal infrared camera was employed in this thermal image collection system. The Trimble BD970 GNSS (Global Navigation Satellite System) OEM (Original Equipment Manufacturer) board was also carried on the quadcopter UAV to gather dual-frequency GNSS observations in order to determine the flying trajectory data by using the Post-Processed Kinematic (PPK) technique; this will be used to establish the position and orientation of collected thermal images with less ground control points (GCPs). The digital surface model (DSM) and thermal orthoimages were then produced from collected thermal images. Tests conducted in the Hsiaoyukeng area of Taiwan’s Yangmingshan National Park show that the difference between produced DSM and airborne LIDAR (Light Detection and Ranging) data are about 37% between −1 m and 1 m, and 66% between −2 m and 2 m in the area surrounded by GCPs. As the accuracy of thermal orthoimages is about 1.78 m, it is deemed sufficient for volcanic geothermal monitoring. In addition, the thermal orthoimages show some phenomena not only more globally than do the traditional methods for volcanic geothermal monitoring, but they also show that the developed system can be further employed in Taiwan in the future.

## 1. Introduction

To discover the general characteristics of volcanic activity, different geoscience observations can be employed. Volcanic monitoring can be further conducted using real-time monitoring systems such as seismic observation, volcanic gas analyses, geodesy survey and geothermal measurement. Thus, continuous geothermal measurement [1] plays one of the most important roles in volcanic monitoring at the Taiwan Volcano Observatory-Tatun (TVO). It is obvious that both surface temperature and geothermal gradients will be higher if a magma chamber or post-volcanic activity exists beneath the volcanic area [2]. In fact, volcanic activity could not be well understood without determining the trend of geothermal variations from long-term measurements [3]. Although the current monitoring system used to measure terrain temperature information obtains data with high frequency and high accuracy, the system is expensive and gathers data at only one site. It is also much more difficult to obtain the trend of temperature change globally. Thermal infrared cameras can be used to sense the temperature information of sensed scenes. With the development of UAS, the thermal infrared cameras can be carried on the quadcopter UAV to gather high resolution thermal images in a local area more appropriately. Thermal cameras have been installed on UAS for use in different studies or for other applications. For example, Ambrosia et al. [4] employed a UAS equipped with a thermal camera and a satellite uplink/downlink image data telemetry system to generate near real-time geo-rectification imagery for disaster managers. Berni et al. [5] described the method to obtain quantitative remote-sensing products using a helicopter-based UAS with inexpensive thermal and narrowband multispectral sensors. Miraliakbari et al. [6] produced orthophoto mosaicking using both RGB and thermal infrared images collected from a UAS carrying a Canon EOS 350D (Canon, Amstelveen, The Netherlands) camera and a FLIR SC660 thermal camera (FLIR, Wilsonville, OR, USA). Hartmann et al. [7] discussed the automatic orientation of thermal images acquired from a UAS using photogrammetric approach and artificial ground control points (GCPs). Their tests showed that the position of thermal images was determined with an accuracy of less than ±10 cm, better than that obtained with direct geo-referencing using on-board single-frequency GPS (Global Position System) receiver. Řehák and Pavelka [8] used the UAS for thermal monitoring of dumps.

For geothermal applications using the UAS, Nishar et al. [9,10] used a small and cost effective quadcopter UAS with an FLIR Tau 320 camera to accurately map the physical and biological characteristics of unique habitats within the Wairakei-Tauhara geothermal field near Taupo, New Zealand. An area of about 700 m^2^ was flown and the images mosaic-ed using Pix4D (Pix4D, Lausanne, Switzerland) and ArcGIS 10.2 (ESRI, San Diego, CA, USA). The orthorectified image with 0.5 cm pixels size was used to show hot thermal anomaly. Based on the results of the studies by Nishar et al. [9,10], it is believed that remote sensing using a UAS could revolutionize the exploration of geothermal energy and the associated ecosystems, in spite of the battery life, navigation capabilities, and flight regulations limits. Harvey et al. [11] demonstrated that no technical barriers stand in the way of using UASs to produce accurate thermal maps in large and inaccessible geothermal areas. A 2.2 km^2^ georeferenced, temperature-calibrated thermal orthophoto of the Waikite geothermal area in New Zealand was presented in their study. Over a period of about 2 weeks, nearly 6000 thermal images were collected. This was also the first test to generate an orthomosaic image of a large geothermal area using a UAS equipped with a thermal camera. Recent advances in UAS technology, combined with lightweight thermal cameras provide a new tool for volcanic monitoring. Amici et al. [12] presented the preliminary results of a test on an Italian mud volcano using a multi-rotor aircraft in a hexacopter UAS. The UAV flew above the Le Salinelle mud volcano located on the lower South West flank of the Mt. Etna volcano. This was a representative site where activity proved to be related to the early stages of magma accumulation within the volcano. In their study, the in-flight measurements were used to cross-validate with in situ collection of thermal information and from independent temperature measurements of mud/water contemporaneously. Mori et al. [13] performed volcanic plume surveys using a multirotor UAS to collect data concerning plume gas composition, sulfur dioxide flux, temperature data, and a particle sampling of Japan’s Mt. Ontake on 20 and 21 November 2014, and on 2 June 2015. Together with the results of manned helicopter and aircraft observations, it was concluded that the plume of Mt. Ontake was not being directly emitted from the magma, but rather, was being influenced by the hydrothermal system.

Specifically, Mori et al. [13] used a multirotor UAS to collect volcanic gas for study in volcanology. However, date back to 2007, McGonigle et al. [14] has employed a helicopter UAS carrying an ultraviolet spectrometer, an infrared spectrometer, and electrochemical sensor for the measurements of volcanic gases at La Fossa crater, Vulcano, Italy, during April 2007. Xi et al. [15] utilized a low-cost UAS carrying gas sensors to collect data for volcanic gas composition and flux analysis of Turrialba volcano, Costa Rica, during 11–13 March 2013.

However, to date the above-mentioned studies of UAS equipped with thermal cameras for volcanic monitoring that have been reported used only a single thermal image for measurements and without orthorectifying them into thermal orthoimages. Therefore, in this study, the thermal image collection of the quadcopter UAS for volcanic geothermal monitoring was not only developed, but the thermal orthoimages were also produced and evaluated. This system was equipped with an XM6 thermal infrared camera. Additionally, the Trimble BD970 GNSS (Global Navigation Satellite System) OEM (Original Equipment Manufacturer) board was carried on the quadcopter UAS to collect data for determining the flying trajectory data using the Post-Processed Kinematic (PPK) technique, and to support the determination of the position and orientation of collected thermal images. The next sections will introduce the system selection and design; the processing procedures of thermal orthoimage will be described in Section 3, related tests and discussions will be offered in Section 4, and conclusions in Section 5.

## 2. UAS System Selection and Design

**Figure 1:**
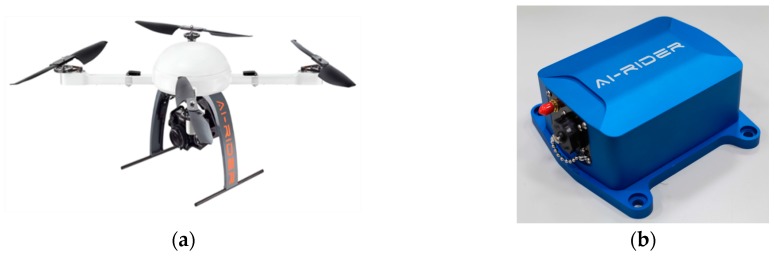
Quadcopter (**a**) Unmanned Aircraft Vehicle (UAV) and (**b**) inertial measurement units (IMU) used in this study. (Source: AI-RIDER Corp.).

The volcanic terrain in Taiwan is of highly variable topography and it leads to extreme environmental conditions. Rotor-craft UASs can take off and land vertically, making them more flexible and appropriate than fixed-wing UASs in flying at low elevations over varied terrain while collecting high-resolution thermal images. For example, Amici et al. [14] used a hexacopter UAS for the preliminary test on an Italian mud volcano, and Mori et al. [15] performed volcanic plume surveys using a multi-rotor UAS. Therefore, the AI-RIDER YJ-1000-QC quadcopter UAV (Figure 1a) provided by AI-RIDER Corp. (Taipei, Taiwan) was selected for use in this study because in steady wind of up to 12 m/s. Thus it overcame not only highly variable topography but also for extreme environmental conditions in Taiwan volcanic terrain. The UAV had GPS and compass functionality, with an air pressure sensor and inertial measurement units (IMU) (Figure 1b) installed. Table 1 tabulates the specifications of the IMU. It is powered by a single battery pack which allows an autonomous flight of 15 min–20 min based on the longevity of the battery and the amount of payload. The maximum payload weight is 2.5 Kg and the maximum ceiling is 500 m. The flight control system auto-stabilizes the drone. It provides manual control using standard radio control, and autopilot navigation using a ground control station. It includes several flight mode configurations; in this study, the waypoint plan is used for surveying in autopilot mode.

**Figure 2:**
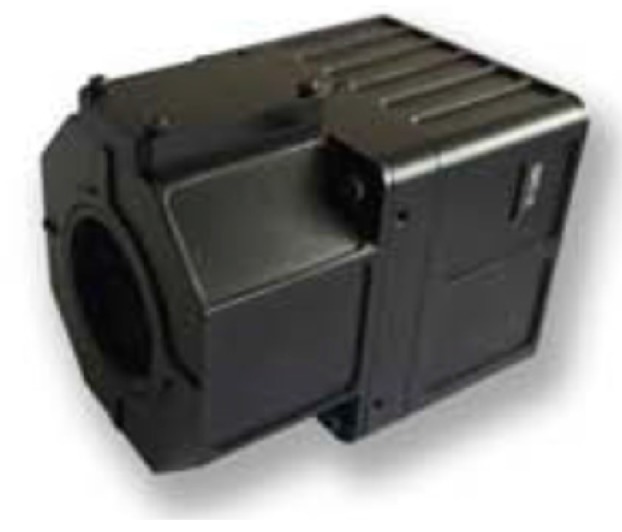
XM6 thermal camera used in this study. (Source: Magnity Electronics Co., Ltd.).

Currently, XM6 (Figure 2) thermal camera is of high resolution, 640 × 480 pixels. The whole sensed data of the infrared spectrum by XM6 can be converted to temperature values with unit °C after corrections, e.g., radiance correction, background temperature correction, optical filter/window correction/atmosphere transmission correction using ThermoScope software provided by Magnity Electronics Co., Ltd. (Shanghai, China). Therefore, the XM6 camera was selected in this thermal image collection system. The specifications of the XM6 thermal camera are tabulated in Table 2. It is composed of 17 μm pitch detectors with a spectral response ranging from 7.5 to 14 μm, and a thermal sensitivity less than 60 mK. The camera has a frame rate of about 25 hertz and can output a PAL (Phase Alternating Line) video.

**Figure 3:**
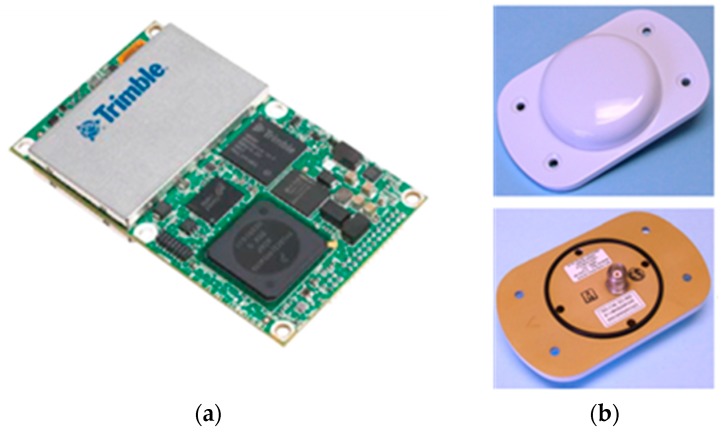
(**a**) Trimble BD970 Global Navigation Satellite System (GNSS) Original Equipment Manufacturer (OEM) board and (**b**) ANTCOM Global Positioning System (GPS) G5 antenna. (Source: Trimble Inc.).

To produce a Digital Surface Model (DSM) and thermal orthoimages, the thermal images should first be positioned and oriented. For volcanic geothermal monitoring, it is difficult to allocate the control targets used to perform the position and orientation of thermal images. Therefore, more accurate trajectory data for the quadcopter UAV is required to support the position and orientation of thermal images with less GCPs. In this study, the Trimble^®^ BD970 GNSS system (Figure 3) including a BD970 GNSS OEM board and the ANTCOM GPS G5 antenna, short for BD970, was adopted. BD970 is a compact multi-constellation receiver capable of delivering centimeter accuracy for a variety of applications. Additionally, the receiving rate for most GNSS receivers is limited to 1 Hz; however the BD970 can collect data at a rate of 50 Hz. Therefore, it was adopted for this study and installed on the UAV for high frequent GNSS data collection. This allowed the GNSS observations corresponding to each thermal image acquisition to be obtained; the collected frequency of GNSS original observations was set to 10 Hz.

## 3. Processing Approach

Before thermal images can be used for orthorectification, the camera parameters and the position and orientation of thermal images should be determined. Section 3.1 describes the approach for camera calibration. Data collection will be described in Section 3.2. Finally, the approach to generating thermal orthoimages will be depicted in Section 3.3.

### 3.1. Camera Calibration Approach for XM6

This study used the in-flight camera calibration approach [16], known as analytical self-calibration, to calibrate the camera. It is a variation of the field method [16]. In this approach, the UAS carrying the camera makes multiple passes in different directions to capture high overlap thermal images, i.e., 80% endlap and 60% sidelap, over the test site. Based on a high number of redundant measurements of natural points from image matching in the thermal images, calibration parameters can be calculated. The in-flight method can also be generalized to the point where calibration parameters are determined in conjunction with the position and orientation of thermal images taken during the actual job.

### 3.2. Data Collection

To produce thermal orthoimages with higher resolution for further analysis, 15 cm is adopted as the maximum ground sampling distance (GSD). Combining the requirements of in-flight camera calibration, high overlap thermal images with high ground resolution, i.e., 80% endlap and 60% sidelap, as well as being based on both the 17 μm pixel size and the 25 mm focal length, the flying height is designed to be about 220 m above the average ground elevation. The coverage of one thermal image is about 72 m × 96 m. Therefore, in designing the UAS flying waypoints, the distance between two exposure stations is about 14 m, and the distance between two strips is about 38 m 

**Figure 4:**
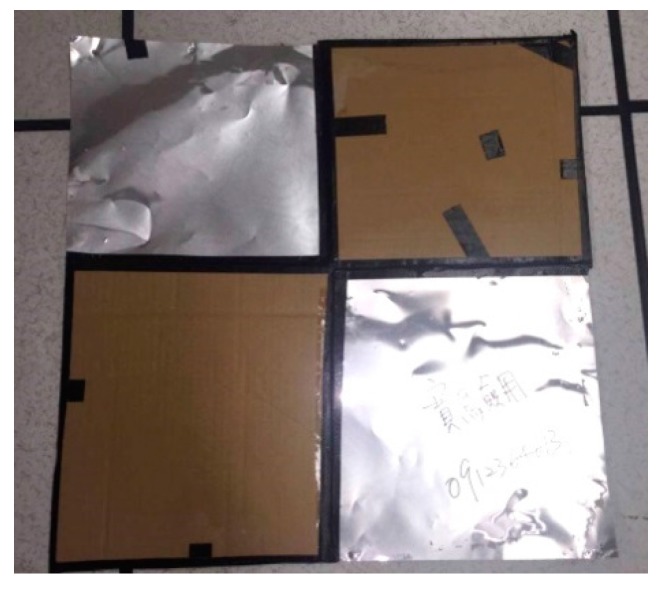
Aluminum target designed for control or check points.

Since the ground resolution is about 15 cm × 15 cm for one pixel, the minimum size of control or check points should be 60 cm × 60 cm for identification. This study used targets with 60 cm × 60 cm as control or check points for ensuring clear identification. Additionally, the 3-D coordinates of control or check points were surveyed by real-time kinematic (RTK) GPS techniques [17]. According to the characteristics of thermal cameras, aluminum is used as the material of control targets or check targets with their size identified clearly in the thermal images. The target is designed as Figure 4, where two quadrants are covered by thick pieces of cardboard in order to locate the point in the thermal images.

In this study, the Trimble BD970 GNSS OEM board was carried aboard the quadcopter UAS to collect dual-frequency GNSS original observations to determine the flying trajectory using the Post-Processed Kinematic (PPK) technique. This supports the position and orientation of collected thermal images, called GNSS-supported position and orientation. Therefore, the GNSS receiver should be put on one ground station, known as the base station, with known 3-D coordinates. The corresponding frequency for receiving data is set as 10 Hz for subsequent calculation.

### 3.3. Generation of Thermal Orthoimages

#### 3.3.1. GNSS-Supported Position and Orientation

##### Establishing a Precise Flying Trajectory

After dual-frequency GNSS original observations were collected from the ground station and UAS, the precise flying trajectory data was determined using the PPK technique to support the position and orientation of the collected thermal images. The algorithm for the PPK is the same as the algorithm used in real-time kinematic (RTK) [17]. However, PPK can use more sophisticated approaches, usually resulting in a more precise position described in three dimensions.

##### GNSS-Supported Position and Orientation of Thermal Images

GNSS-supported position and orientation of the thermal images was determined using a self-calibration method (see Section 3.1). This method is based on bundle adjustment, referred to as self-calibration aerial triangulation (AT) in this study. Before GNSS-supported self-calibration AT determines the position and orientation of the thermal images, the GNSS observations, i.e., (E, N, H) corresponding to each thermal image should be extracted further from the precise flying trajectory data (see Section Establishing a Precise Flying Trajectory) based on the recorded exposure time of each thermal image.

In this study, because the UAV hovered for about 2 s to acquire each thermal image and the smallest unit of recorded time was 1 s, the average of ten GNSS observations in 1 s based on the recorded time for each thermal image was calculated as the GNSS observation. Subsequently all GNSS observations with their corresponding standard deviations were employed to support the orientation and position of the thermal images using self-calibration AT.

Additionally, GNSS observation corresponding to each thermal image is not consistent with the perspective center of each thermal image. Therefore the offset between the thermal image perspective center and GPS antenna center, called the GPS antenna offset [16], should be resolved. In traditional aerial photogrammetry, drift parameters were used to decrease the influence of system errors caused by the GPS antenna-camera offset [16]. IMU instruments could record the angle of yaw, roll and pitch of the UAV used in this study. The resolution and accuracy of attitude of the UAS was 0.1 and 0.5 degrees, respectively. Even though the accuracy of the recorded angle of yaw, roll and pitch was not highly precise, the influence was less than 1 cm as the offset was only 30 cm. Therefore, the offset of the GPS antenna in the *x*, *y*, *z* coordinate of the IMU coordinate system could be converted into the E, N, and H coordinate components based on the angle of yaw, roll and pitch recorded by the IMU. GNSS observations could then be reduced to the thermal image perspective center.

According to the above-mentioned facts, two kinds of GNSS observations were obtained: the non-reduced GNSS observation and the reduced GNSS observation. The corresponding weight of these GNSS observations was set according to their corresponding standard deviations while performing GNSS-supported self-calibration AT. Moreover, the single-frequency GNSS observations from the Fight Control System (FCS) based on a single-frequency GNSS receiver on an UAV was also used to perform GNSS-supported self-calibration AT in order to investigate the accuracy of GNSS-supported self-calibration AT with different kinds of GNSS observations. The above three tests would not use any GCPs in order to verify the feasibility of our idea to reduce GNSS observations into the thermal image perspective center based on IMU records for GNSS-supported self-calibration AT. Therefore, the Root Mean Square Error (RMSE) of check points in E, N, and H coordinates of three different GNSS-supported self-calibration AT without GCPs will be presented. After that, the RMSE of check points of GNSS-supported self-calibration AT using the reduced GNSS observation with four GCPs on the corners of test area will be presented in order to discuss the achievable accuracy of our proposed approach. Due to the difficulty of setting up and surveying the GCPs in volcanic areas, the used four GCPs were selected and measured from historical stereo images.

Therefore, four GNSS-supported position and orientation were performed with different control strategies: (1) single-frequency GNSS observations from a single-frequency calculation as air controls; (2) non-reduced GNSS observations from dual-frequency calculation as air controls; (3) reduced GNSS observations from dual-frequency calculation as air controls; and (4) reduced GNSS observations from dual-frequency calculation as air controls with four GCPs from historical aerial stereo images on the corners of the test area. The software used for GNSS-supported self-calibration AT was Pix4DMapper.

#### 3.3.2. Generation of DSM and Thermal Orthoimages

After the position and orientation of thermal images were determined, DSM was also produced using the Pix4DMapper software. The results of DSM was verified by airborne LiDAR data produced on 13 May 2012 by the comparison of each grid cell with dimensions 0.5 m × 0.5 m. The size of each grid cell was determined based on the point density of the airborne LiDAR data, 4 pt/m^2^. The height of each grid was simply calculated by averaging the heights of all points in each grid. This quantitative analysis was performed by the 3-D spatial analysis tools, subtraction of raster math, in ArcGIS software. Raster size corresponded to the grid size. Thermal orthoimages were then generated based on the DSM using the Pix4DMapper software by setting the cell size. The accuracy of the thermal orthoimages were evaluated by the RMSE of check points in the E, N, and H coordinate components, respectively. Meanwhile, the orthoimages were further used to investigate the volcanic geothermal activities in the test area.

## 4. Results and Discussions

**Figure 5:**
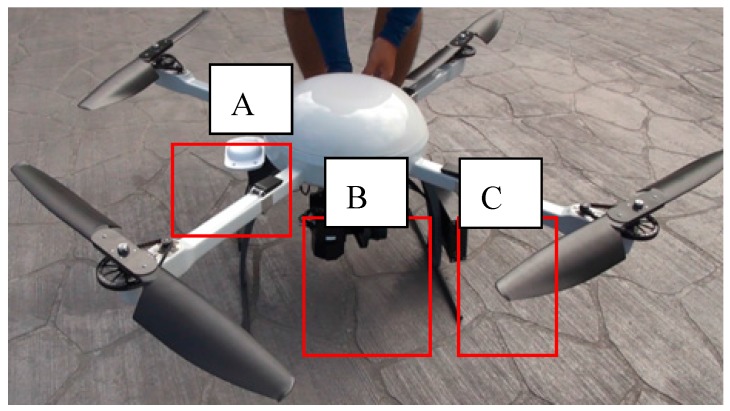
UAV established in this study. (**A**): the antaean; (**B**): XM6 thermal camera; (**C**): BD 970 GNSS OEM board.

The established UAV in this study is shown as Figure 5.

### 4.1. Study Test Area and Waypoint Design

**Figure 6:**
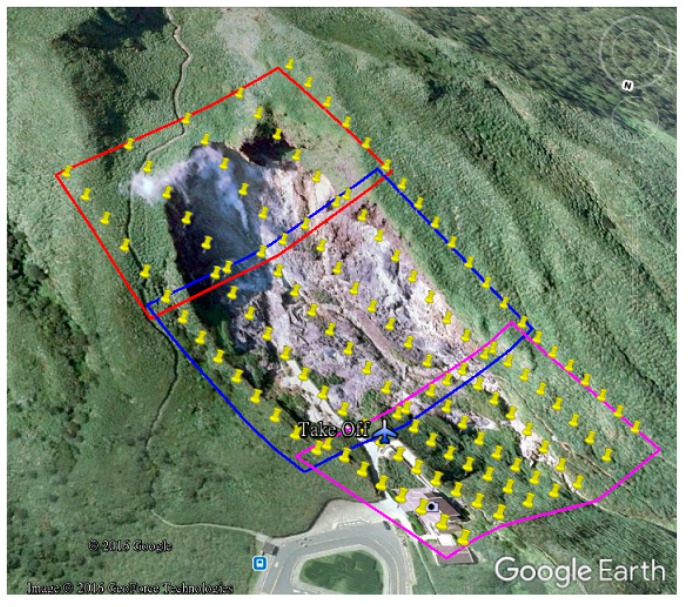
Study area at Hsiaoyukeng in the Tatun volcano group.

On the island of Taiwan, located at the earthquake zone of the western Pacific Ring of Fire, the Tatun volcano group (TVG) is the only one still displaying significant volcanic activity even though scattered volcanic rocks have been identified. Not only is it the biggest volcanic group, but it is also still active [18,19,20,21,22,23,24]. According to recent reports [22,25], the last eruption was within the last thousand years in the TVG. Thus, the possibility of a future volcanic eruption cannot be ruled out. The TVG is composed of more than 20 volcanoes, including Mts. Chishin, Tatun, Huangdra and Shamon. Volcanic geothermal activity is still very strong and a number of fumaroles and hot springs are present. One of the strongest geothermal activities is located at Hsiaoyukeng. Therefore, we have selected this area (ca. 0.7 hectares) as the study area, not only because of significant geothermal activity, but also because of the opportunity to demonstrate the advantages of Rotor-craft UAS in an area with highly variable topography. Figure 6 shows the study test area. Three distinct areas are designated with the UAV flying 220 m above average ground height to acquire thermal images with about 15 cm GSD. According to Section 3.2, the waypoints are designed as shown in Figure 6. The selected site for the vertical takeoff and landing is indicated with the airplane symbol in Figure 6.

### 4.2. Data Collection

**Figure 7:**
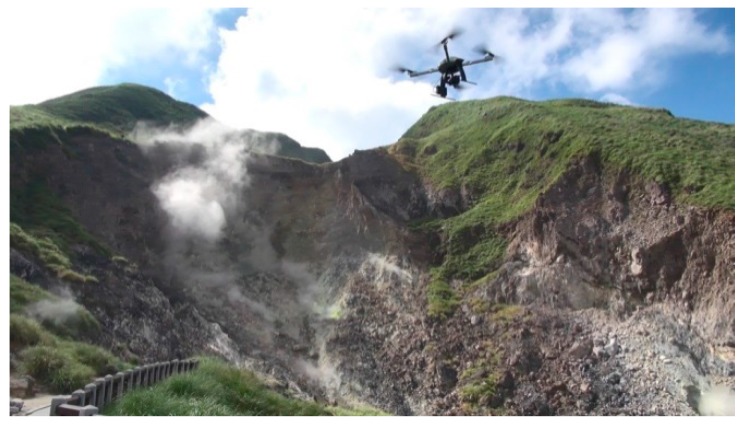
In situ situation of UAV flying.

**Figure 8:**
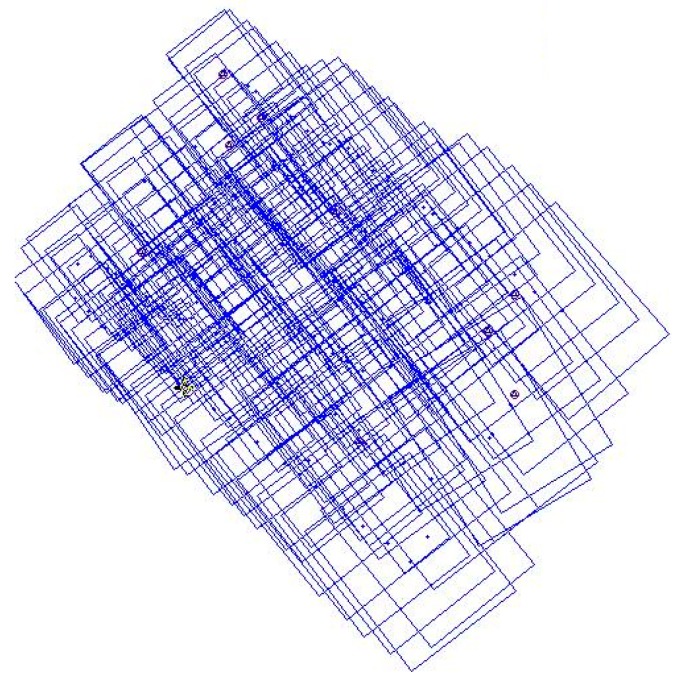
The footprint of used thermal images in this study.

**Figure 9:**
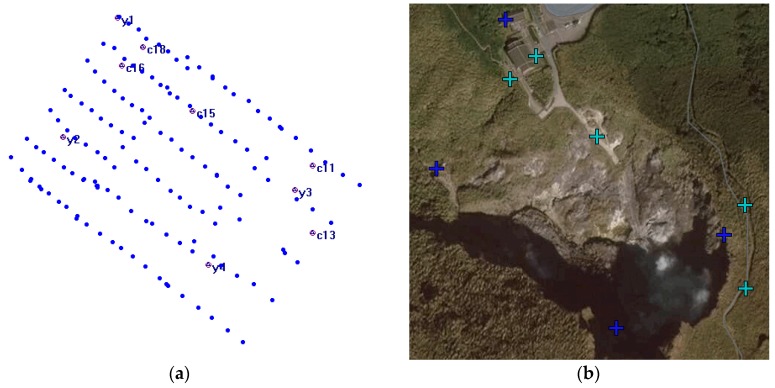
Ground control points (GCPs) and Check Points of GNSS-supported position and orientation of thermal images. GCPs: y1, y2, y3, y4 by stereo measurements from historic aerial stereo images; Check Points: 11, 13, 15, 16, 18 by the real-time kinematic (RTK) GPS Surveying method. (**a**) Locations of control points, check points and image centers, (**b**) Locations of control points, check points overlaied with Google Image.

The quadcopter flew above each area (Figure 6) continuously acquiring thermal images for about 15 min during each flight on 10 August 2016. The receiving rate of GNSS original observations in this study is set as 10 Hz. It was about 7:00 a.m. when we arrived at the study test area and the weather was cloudy and windy. It took about 4 h to acquire 216 thermal images. Figure 7 shows that the acquisition system worked properly although the topography was relatively extreme. The footprint of the 145 thermal images acquired for subsequent processing is shown in Figure 8. The thermal images display an 80% end lap and 60% side lap. The coverage of each acquired thermal image is about 72 m × 96 m with total coverage of about 68,000 m^2^. Due to the limits of the terrain, it was difficult to setup the GCPs. As a result, only four GCPs (see Figure 9) could be measured from the historical aerial stereo images, proving the importance of GNSS-supported position and orientation. Additionally, five check points (the locations as shown in Figure 9) designed as Figure 4 were set up and surveyed using the RTK GPS surveying method to verify the accuracy of the GNSS-supported position and orientation. The base-station was located about two kilometers from the study test site in order to survey check points by the RTK GPS surveying method, and to determine the trajectory data using the PPK technique.

### 4.3. Generation of Thermal Orthoimages

#### 4.3.1. GNSS-Supported Position and Orientation

##### Establishing a Precise Flying Trajectory

As mentioned in Section 3.3.1, the parts of the calculated precise GNSS observations by PKK are shown in Table 3. Table 3 shows the precision of GNSS observations by PKK are about (0.8 cm, 0.7 cm, 2.0 cm) in E, N, H coordinate components.

##### GNSS-Supported Position and Orientation of Thermal Images

**Figure 10:**
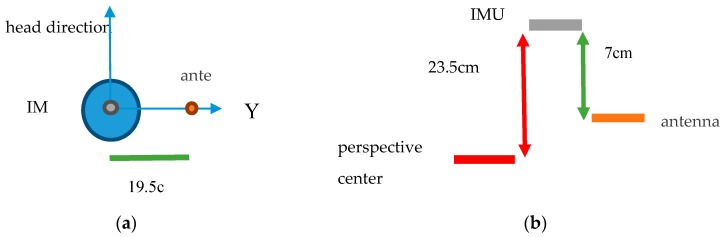
The offset illustration between IMU center and the antenna center. (**a**) Top view; (**b**) Side view.

According to the description in Section 3.3.1, the GNSS observations corresponding to each thermal image needed to be processed further based on the recorded exposure time of each thermal image. In this study, the average of ten GNSS observations in 1 s and the standard deviation for each thermal image was calculated as the GNSS observation and corresponding error; all the GNSS observations with their corresponding standard deviations were then employed in self-calibration AT to support the orientation and position of thermal images. In this study, the GNSS observations of one test were reduced to the center of perspective center by the offset between IMU and antenna (see Figure 10) and the recorded roll, yaw and pitch data from IMU.

As described in Section 3.3.1, four GNSS-supported position and orientation were performed using different control strategies: (A) single-frequency GNSS observations as air controls(i.e., the GNSS observations from the Flight Control System (FCS) on an UAV); (B) non-reduced GNSS observations from dual-frequency calculation as air controls; (C)reduced GNSS observations from dual-frequency calculation as air controls; and (D) reduced GNSS observations from dual-frequency calculation as air controls with four GCPs from historical aerial stereo images. The software employed was Pix4DMapper (Pix4D, Lausanne, Switzerland). The corresponding error of these GNSS observations was set according to their corresponding standard deviations in the E, N, and H coordinate components while performing GNSS-supported self-calibration AT. In particular, in control strategy B, using non-reduced GNSS observations as air controls, the standard deviation in E-N planimetry is its corresponding standard deviations plus 0.2 m, and its corresponding H standard deviations plus 0.2 m in order to overcome the offset problem between the antenna center and perspective center.

The 5 check point RMSE of GNSS-supported self-calibration AT based on different control strategies are shown in Table 4. The distribution of check points is illustrated as Figure 9. The results in Table 4 show that the accuracy attained using reduced GNSS observations as air controls is more precise than the accuracy of the other two control strategies A and B; however, the RMSE in E, N, and H coordinate were 6.5 m, 7.5 m, 4.8 m. With four GCPs from historical aerial stereo images added, the horizontal and vertical accuracy could be promoted to 2.1 m and 3.1 m; it is believed that this is already sufficient for volcanic geothermal monitoring applications.

**Figure 11:**
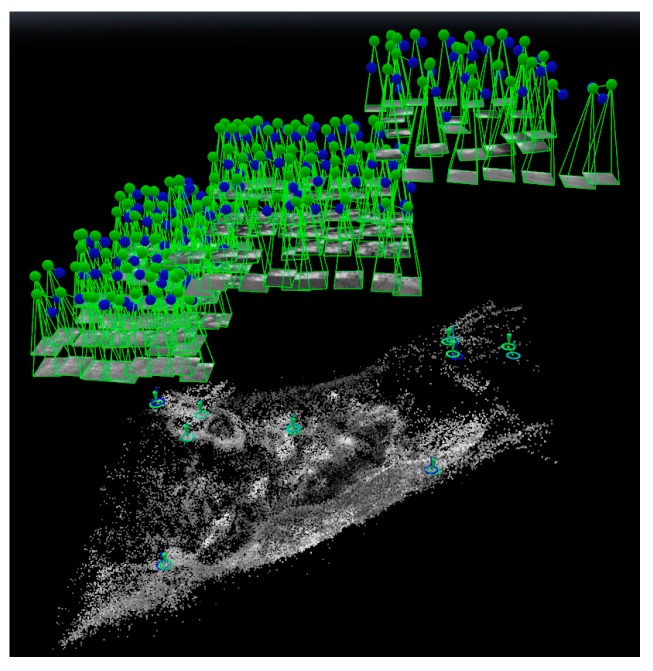
The result of GNSS-supported self-calibration AT using control strategy D.

Figure 11 shows the result of position and orientation of thermal images determined by GNSS-supported self-calibration AT by reduced GNSS observations with four GCPs as controls.

#### 4.3.2. Generation of DSM and Thermal Orthoimages

**Figure 12:**
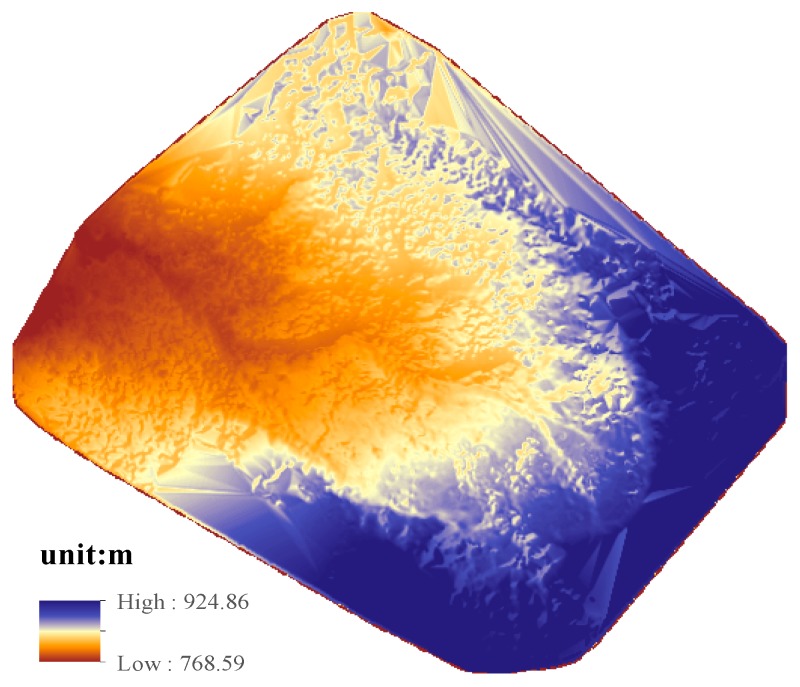
The generated digital surface model (DSM) image.

**Figure 13:**
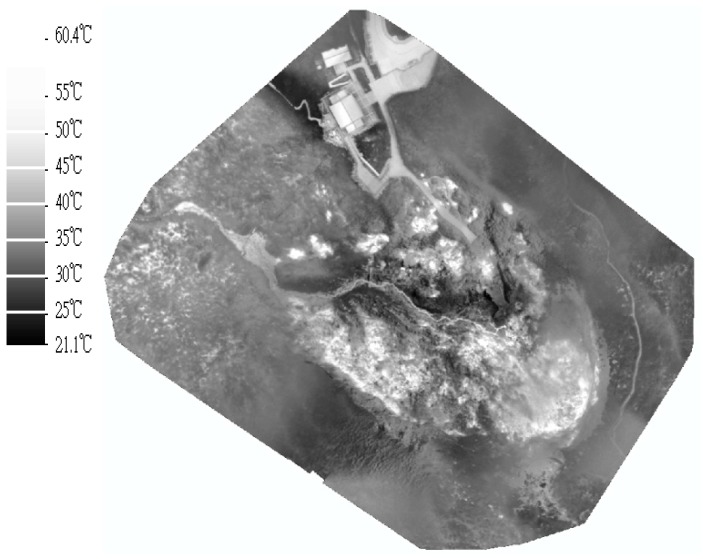
Thermal orthoimage.

**Figure 14:**
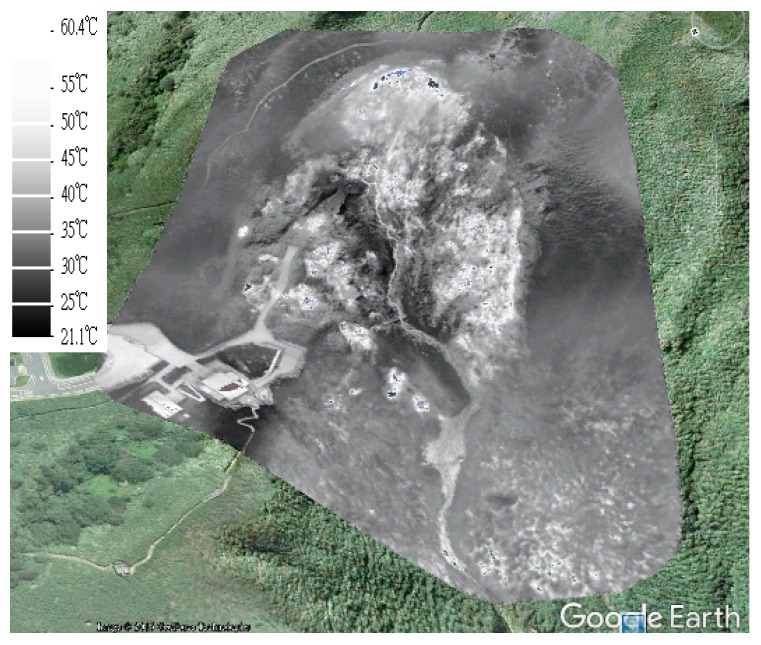
The overlay of thermal orthoimage and Google Earth.

After the position and orientation of thermal images were determined, the densified point cloud could be generated by the Pix4DMapper software. The DSM could be generated from the point clouds, and the thermal orthoimages could also be generated using DSM without manual editing. Figure 12 and Figure 13 show the DSM and the thermal orthoimage with the grid ground resolution at 15 cm × 15 cm. Figure 14 shows the thermal orthoimage overlaid with Google Earth. The whole sensed data of the infrared spectrum by XM6 was digital radiant values. They are converted to temperature values with unit °C based on an estimated emissivity of the sensed body (sulfur surface) about 0.92 [26] and 20 °C environment temperature using ThermoScope software provided by Magnity Electronics Co., Ltd. (Shanghai, China).

**Figure 15:**
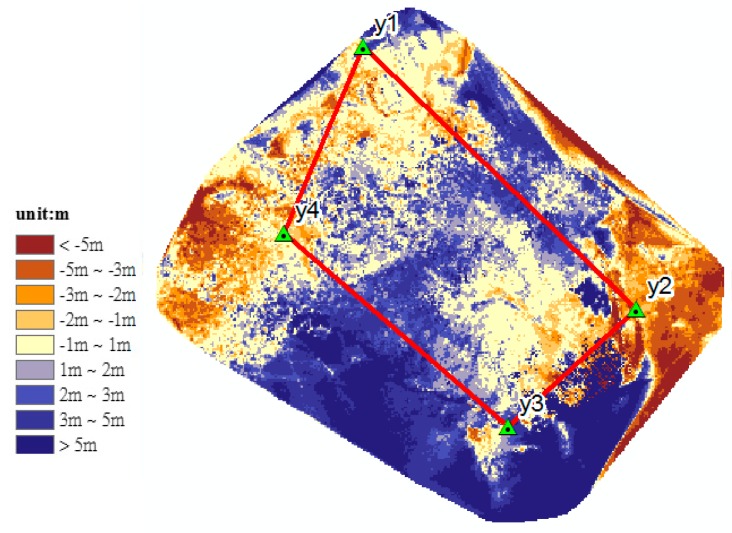
The illustration of quantitative analysis for thermal DSM (thermal DSM elevations minus LiDAR DSM).

Because the thermal orthoimage is highly related to the quality of DSM, the quantitative analyses were first done with airborne LiDAR data collected in 13 May 2012. Figure 15 shows the quantitative analysis. This quantitative analysis was performed by the 3-D spatial analysis tools, subtraction of raster math, in ArcGIS software. Raster size was set to 0.5 m to perform this analysis of two DSMs. In the area surrounded by the GCPs (y1, y2, y3 and y4) (see red quad rectangles in Figure 15), the result obtained from thermal DSM elevations minus from LiDAR DSM shows that the difference between produced DSM and airborne LIDAR data is about 36% between −1 m to 1 m (see Table 5) and 66% between −2 m to 2 m and the larger difference about 29% is greater than 2 m (light blue or blue areas) located in grassy areas.

**Figure 16:**
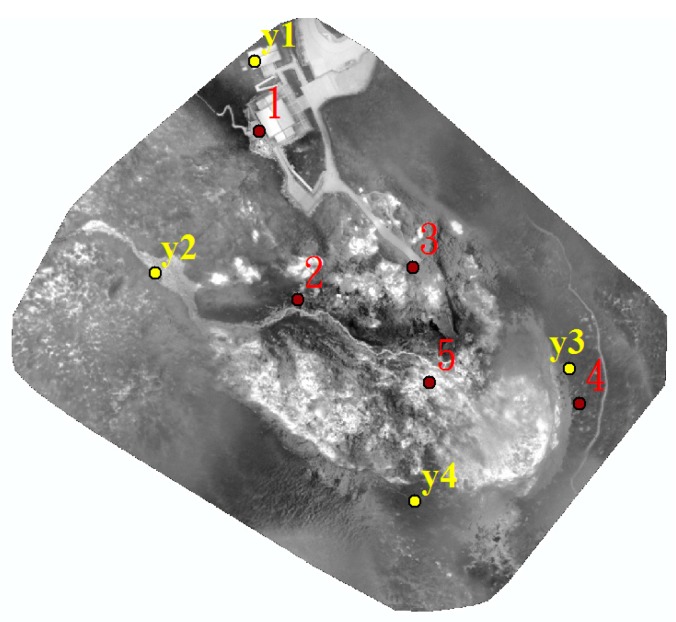
The location of 5 check points for the verification of geometric planimetric accuracy.

For further verification of the geometric accuracy of thermal orthoimages, the even distribution of 5 check points (see Figure 16) from historic aerial stereo images were measured. The planimetric discrepancy of each check point was calculated to check the results of the thermal orthoimages. The checked results are shown in Table 6. These show that the average discrepancy is 1.54 m, maximum discrepancy is 3.17 m, and the minimum discrepancy is 0.71 m. The planimetric accuracy is 1.78 m. The results prove the thermal orthoimage is sufficiently accurate for volcanic geothermal monitoring.

### 4.4. Further Investigation and Discussion

Based on the preliminary results shown in Figure 13 and Figure 14, we can clearly see the general thermal variations on the ground surface. First, a lot of intense geothermal activities, such as fumaroles and hot-springs, can be identified by the white spots. Of particular interest are several isolated white spots around the path connected to the building of the visitor center. Second, all of white spots are basically limited to the subsidence area of Hsiaoyukeng. A white half-ring is shown as roughly following the abandoned cliff, where geothermal activity is stronger than in other areas. Third, it is very interesting to find a thin line, like a creek, among those spots in the middle of the examined area. The line is obviously associated with hot water coming from the fumaroles and hot-springs at higher altitudes. The thin line becomes grayer as it flows to the lower altitudes because heat is gradually being lost on the surface. In summary, the identification of these general patterns provides a powerful tool for monitoring any future volcanic activity.

## 5. Conclusions and Suggestion

In this study, the quadcopter UAS was developed for the collection of thermal images to be used in volcanic geothermal monitoring in Taiwan to overcome the difficult terrain with highly variable topography and extreme environmental conditions. An XM6 thermal infrared camera was used in this thermal image collection system. Additionally, the Trimble BD970 GNSS OEM board was carried on the quadcopter UAV to collect the precise flying trajectory data for determining the position and orientation of thermal images with less ground control points (GCPs). The experiment was successfully performed in the Hsiaoyukeng area of Taiwan’s Yangmingshan National Park on 10 August 2016. The system performed very well under extreme environmental conditions. The PPK GPS technique was used to determine the flying trajectory data for collecting highly precise GNSS observation data, (ca. 0.7 cm, 0.8 cm, 2 cm) standard deviation in E, N, H directions, to support the position and orientation of collected thermal images with less GCPs. In this study, the GPS antenna offset problem was solved by introducing the yaw, roll and pitch data recorder by IMU. GNSS observations could be reduced to the center of thermal image perspective center by the recorded yaw, roll and pitch data. The tests show that the position and orientation of thermal images determined using reduced GNSS observations are superior to those produced using non-reduced GNSS observations without any GCPs. Meanwhile the RMSE of GNSS-supported position and orientation, i.e., self-calibrating AT, by using reduced GNSS observations from dual-frequency calculations and four GCPs from historical aerial stereo images as controls are 1.6 m, 1.4 m, and 3.1 m in E, N, H coordinate components, respectively. Even the GPS antenna offset problem was solved by this simple approach; the result is suitable for volcanic geothermal monitoring. After determining the position and orientation of thermal images, the DSM and thermal orthoimages were generated. The accuracy of DSM was checked quantitatively by airborne LIDAR data; it is shown that the difference between produced DSM and airborne LIDAR data is about 36% between −1 m to 1 m in the area surrounded by the GCPs. Additionally, the accuracy of the thermal orthoimage is about 1.78 m; this also shows that the generated thermal orthoimage is suitable for subsequent application.

From the thermal orthoimages, the fact that some phenomena cannot be observed using the traditional methods is clearly presented more globally, even though temperature correction was not done in this study. It also proves that a UAS can support monitoring operations in certain difficult and dangerous circumstances. A handheld FLIR thermal camera will be used for cross-comparison with the data acquired during a future flight experiment, and the approach to temperature correction of orthorectified thermal images will be further investigated in a future study. Moreover, our proposed UAS and approach might find application in the future monitoring of volcanic activity in Taiwan, i.e., the orthoimages of two different periods will be used to investigate the temperature change for volcanic monitoring application.

## Figures and Tables

**Table 1:** Specifications of the IMU. (Source: AI-RIDER Corp.).

3-Axis Rate Gyro
Sensing Range ≥ ±250°/s
Resolution Per-axis ≥16 bit
Data Update Rate ≥100 Hz
3-axis G-sensor
Sensing Range ≥ ±6 g
Resolution Per-axis ≥16 bit
Data Update Rate ≥100 Hz
3-axis m-sensor
Sensing Range ≥ ±6 gauss
Resolution ≤ 0.195 milli gauss/count
Data Update Rate ≥100 Hz

**Table 2:** Specifications of the XM6 thermal camera. (Source: Magnity Electronics Co., Ltd.).

**Detector**	
Detector type	uncooled FPA
Spectral band	7.5~14 μm
Resolution	640 × 480 pixels
Pixel size	17 μm
Max frame rate	25 Hz
**Measurement**	
Temperature measurement range	−20–150 °C/−20–300 °C
Accuracy	±2 °C or ±2%
Thermal sensitivity(NETD)	<60 mk
**Lens**	
Focal length	25 mm
Field of view	25° × 19°
Spatial resolution	0.68 mrad

**Table 3:** Part listing of GNSS observations by PPK.

Date	Time (s)	E (m)	N (m)	H (m)	STD_E (m)	STD_N (m)	STD_H (m)
10 August 2016	10:37.7	305,081.615	2,785,308.749	995.531	0.007	0.007	0.014
10 August 2016	10:37.8	305,081.599	2,785,308.751	995.531	0.007	0.007	0.014
10 August 2016	10:37.9	305,081.591	2,785,308.746	995.533	0.005	0.005	0.010
10 August 2016	10:38.0	305,081.579	2,785,308.751	995.538	0.006	0.006	0.012
10 August 2016	10:38.1	305,081.566	2,785,308.755	995.545	0.006	0.006	0.011
10 August 2016	10:38.2	305,081.562	2,785,308.754	995.545	0.009	0.009	0.019
10 August 2016	10:38.3	305,081.551	2,785,308.748	995.575	0.007	0.007	0.014
10 August 2016	10:38.4	305,081.550	2,785,308.747	995.587	0.007	0.007	0.014
10 August 2016	10:38.5	305,081.538	2,785,308.743	995.601	0.007	0.007	0.014
10 August 2016	10:38.6	305,081.538	2,785,308.733	995.625	0.004	0.004	0.008
10 August 2016	10:38.7	305,081.531	2,785,308.728	995.657	0.008	0.008	0.016
10 August 2016	10:38.8	305,081.529	2,785,308.719	995.690	0.005	0.005	0.010
10 August 2016	10:38.9	305,081.527	2,785,308.710	995.740	0.007	0.007	0.014
10 August 2016	10:39.0	305,081.521	2,785,308.702	995.784	0.008	0.007	0.015
10 August 2016	10:39.1	305,081.512	2,785,308.681	995.825	0.005	0.005	0.010
10 August 2016	10:39.2	305,081.509	2,785,308.676	995.867	0.007	0.007	0.015
10 August 2016	10:39.3	305,081.499	2,785,308.666	995.923	0.007	0.007	0.014
10 August 2016	10:39.4	305,081.485	2,785,308.650	995.980	0.005	0.005	0.010
10 August 2016	10:39.5	305,081.467	2,785,308.639	996.017	0.008	0.008	0.015
10 August 2016	10:39.6	305,081.447	2,785,308.628	996.053	0.009	0.009	0.017
10 August 2016	10:39.7	305,081.432	2,785,308.621	996.111	0.007	0.007	0.014

**Table 4:** The accuracy of GNSS-supported self-calibration aerial triangulation (AT) based on different control strategies.

Control Strategies and The Corresponding Observation Errors	RMSE (unit: m) of Check Points			
	E	N	E_N	H
A. Single-frequency GNSS observations from single-frequency calculation as air controls (GNSS observation error: 5 m in planimetry, 5 m in H height)	25.2	20.5	32.5	13.9
B. Non-reduced GNSS observations from dual-frequency calculation as air controls (GNSS observation error: corresponding planimetric standard deviations +0.2 m in planimetry, corresponding H standard deviations +0.2 m in height)	7.7	9.0	11.8	9.1
C. Reduced GNSS observations from dual-frequency calculation as air controls (GNSS observation error: corresponding planimetric standard deviations in planimetry, corresponding H standard deviations in height)	6.5	7.5	9.9	4.8
D. Reduced GNSS observations from dual-frequency calculation as controls with four GCPs (GNSS observation error: corresponding planimetric standard deviations in planimetry, corresponding H standard deviations in height) GCPs: E-N planimetry: 0.28 m; H: 0.3 m)	1.6	1.4	2.1	3.1

**Table 5:** The difference statistics between two DSMs generated from thermal images and airborne LiDAR.

Difference	No. of Grid Cells	Percentage
<−5 m	85	0.40%
−5 m~−3 m	228	1.08%
−3 m~−2 m	643	3.06%
−2 m~−1 m	1872	8.90%
−1 m~1 m	7685	36.52%
1 m~2 m	4433	21.07%
2 m~3 m	3450	16.40%
3 m~5 m	2168	10.30%
>5 m	478	2.27%
Total	21,042	100.00%

**Table 6:** The checked data for the geometric accuracy of thermal orthoimages.

Check Point	Discrepancy of Point Location Between Check Points (Unit: m)
1	0.71
2	1.18
3	0.85
4	3.17
5	1.77
Average	1.54
RMSE	1.78
